# Emergence of dysfunctional neutrophils with a defect in arginase-1 release in severe COVID-19

**DOI:** 10.1172/jci.insight.171659

**Published:** 2024-09-10

**Authors:** Amrita Dwivedi, Aisling Ui Mhaonaigh, Makala Carroll, Bahareh Khosravi, Isabella Batten, Robert Seán Ballantine, Stuart Hendricken Phelan, Laura O’Doherty, Angel Mary George, Jacklyn Sui, Heike C. Hawerkamp, Padraic G. Fallon, Elnè Noppe, Sabina Mason, Niall Conlon, Clíona Ni Cheallaigh, Conor M. Finlay, Mark A. Little, on behalf of the St James’s and Tallaght Trinity Allied Researchers (STTAR) Bioresource

**Affiliations:** 1 Trinity Kidney Centre, School of Medicine, and; 2 Department of Medical Gerontology, Trinity Translational Medicine Institute, Trinity College Dublin, Dublin, Ireland.; 3 Wellcome Trust, Clinical Research Facility;; 4 Department of Infectious Diseases; and; 5 Department of Immunology, St James’s Hospital, Dublin, Ireland.; 6 School of Medicine, Trinity Biomedical Sciences Institute;; 7 Department of Immunology, Trinity Translational Medicine Institute; and; 8 Department of Critical Care, Tallaght University Hospital, Trinity College Dublin, Dublin, Ireland.; 9 The STTAR Bioresource is detailed in Supplemental Acknowledgments.

**Keywords:** COVID-19, Immunology, Neutrophils

## Abstract

Neutrophilia occurs in patients infected with SARS-CoV-2 (COVID-19) and is predictive of poor outcomes. Here, we link heterogenous neutrophil populations to disease severity in COVID-19. We identified neutrophils with features of cellular aging and immunosuppressive capacity in mild COVID-19 and features of neutrophil immaturity and activation in severe disease. The low-density neutrophil (LDN) number in circulating blood correlated with COVID-19 severity. Many of the divergent neutrophil phenotypes in COVID-19 were overrepresented in the LDN fraction and were less detectable in normal-density neutrophils. Functionally, neutrophils from patients with severe COVID-19 displayed defects in neutrophil extracellular trap formation and reactive oxygen species production. Soluble factors secreted by neutrophils from these patients inhibited T cell proliferation. Neutrophils from patients with severe COVID-19 had increased expression of arginase-1 protein, a feature that was retained in convalescent patients. Despite this increase in intracellular expression, there was a reduction in arginase-1 release by neutrophils into serum and culture supernatants. Furthermore, neutrophil-mediated T cell suppression was independent of arginase-1. Our results indicate the presence of dysfunctional, activated, and immature neutrophils in severe COVID-19.

## Abstract 2

Here we show altered neutrophil phenotypes and function is a feature of COVID-19 infection and that immature low-density neutrophils correlate with disease severity.

## Graphical abstract

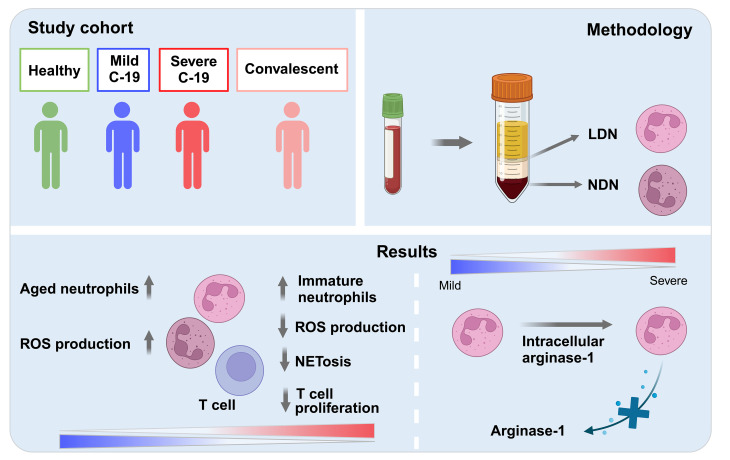

## Introduction

COVID-19 is characterized by neutrophilia and an increased neutrophil-to-lymphocyte ratio, which are independent risk factors for severe disease (1, 2). The associated pneumonic process is neutrophil rich (3). In COVID-19, the extensive release of neutrophil extracellular traps (NETs) is a key contributor to the characteristic immune-mediated thrombosis (4–8), which affects not only the lungs but also the liver and kidney (9).

Low-density neutrophils (LDNs) are a subpopulation of neutrophils found at high frequency in several inflammatory diseases, including cancer, sepsis, and autoimmunity (10–14). During density gradient centrifugation of peripheral blood, the LDNs migrate with the peripheral blood mononuclear cell (PBMC) fraction, a characteristic that distinguishes the LDNs from most neutrophils in the circulation that sediment in the normal-density neutrophil (NDN) fraction. LDNs are a heterogenous population of mature and immature neutrophils, with studies reporting contradictory roles of LDN in inflammation. On the one hand, they have been reported to be pro-inflammatory in systemic lupus erythematosus (15, 16), whereas in cancer, they are reported to possess an immunosuppressive phenotype (17, 18).

Single-cell RNA-sequencing analysis of PBMCs from patients with COVID-19 has defined the characteristics of LDN in individuals with severe disease (19). The analysis showed that the LDN fraction was composed of transcriptionally heterogeneous subsets of neutrophils expressing various genes, such as those involved in NET formation, alarmins, and arginase-1 (19). Morrissey et al. reported increased levels of the CD16-intermediate (CD16^int^) subset of LDNs in blood and bronchoalveolar lavage fluid of patients with severe COVID-19, which were associated with increased D-dimer levels and enhanced platelet activation (20). Owing to the elevated expression of NET-associated genes in the LDN fraction, LDNs may contribute to the inflammation and tissue damage seen in severe COVID-19. However, we do not have a full picture of the role of LDNs in COVID-19 or the potential of LDNs as a pathogenic or prognostic marker for the spectrum of disease in patients with COVID-19.

A study by Derakhshani et al. reported increased arginase-1 (*ARG1*) gene expression in the whole blood of patients with COVID-19, suggesting that *ARG1* has the potential to be a useful marker of disease (21). *ARG1* encodes the enzyme arginase-1, which is released from neutrophils during degranulation. The release of arginase-1 from neutrophils can contribute to the resolution of inflammation by converting the amino acid arginine into ornithine and urea, thus reducing the availability of arginine for protein synthesis and the production of nitric oxide (22). During steady state, neutrophils constitutively express arginase-1, which is stored in gelatinase granules (23). In certain disease states, arginase-1 is released extracellularly, including cancer, where solid tumor-infiltrating neutrophils have decreased intracellular arginase-1, suggesting that it has been released into the tumor microenvironment (24).

In this study, we examined how the neutrophil compartment becomes dysregulated during severe COVID-19. We identified the emergence of disease-specific subsets of neutrophils, including an increase in arginase-1 expression in severe COVID-19 that is retained into convalescence.

## Results

### Patient characteristics.

We recruited patients with active (*n* = 79) and convalescent (*n* = 12) COVID-19, healthy controls (*n* = 36) (Table 1), and patients with sepsis (*n* = 29, Supplemental Table 1; supplemental material available online with this article; https://doi.org/10.1172/jci.insight.171659DS1). Patients with active disease had WHO severity scores of 1 (*n* = 11), 2 (*n* = 1), 3 (*n* = 33), 4 (*n* = 16), 5 (*n* = 12), 6 (*n* = 5), and 7 (*n* = 1). These were divided into mild, moderate, and severe based on these WHO scores: mild (score of 1, 2, or 3), moderate (score of 4), severe (score of ≥5). The median interval between the date of positive PCR result and sampling for patients with active disease was 6 days (range, 0–39), while the interval between date of discharge and sampling for convalescent individuals was 78 days (range, 47–254) (Table 1). Detailed participant characteristics are defined in the Supporting Data Values file.

### Phenotypic analysis of whole blood neutrophils revealed distinct neutrophil populations in mild and severe COVID-19.

**Figure 1:**
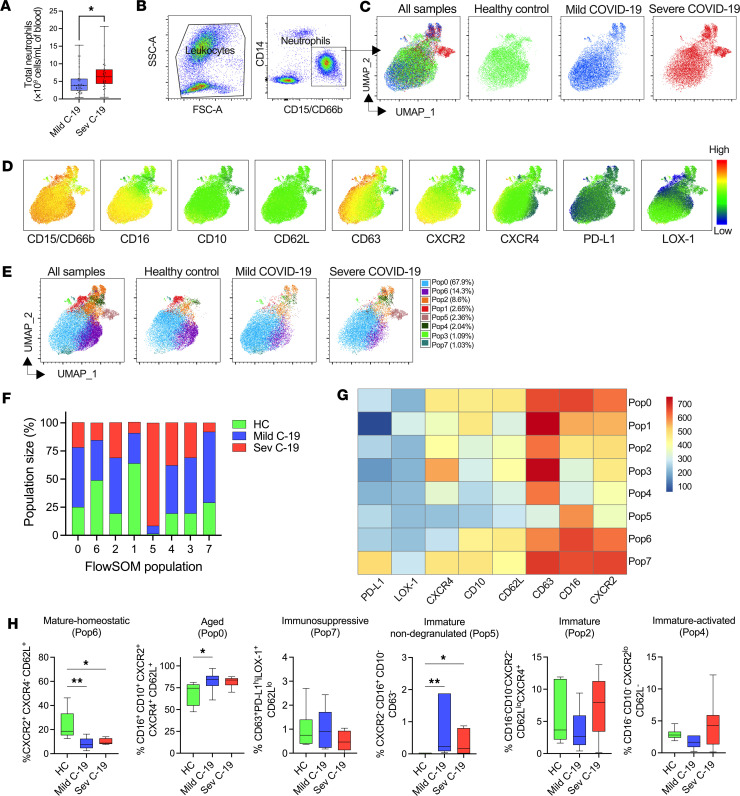
Phenotypic analysis of whole blood neutrophils reveals distinct neutrophil populations in mild and severe COVID-19. Fresh whole blood from healthy controls and patients with COVID-19 was subjected to phenotypic analysis using a combination of markers identifying the activation, maturation, and immunomodulatory status of neutrophils. (**A**) Total neutrophil count from patients with mild (*n* = 41) and severe (*n* = 29) COVID-19. (**B**) Flow cytometric gating strategy for identification of neutrophils in whole blood. (**C**) A total of 63,000 cells concatenated from 6 healthy controls (HC) and 10 patients with mild COVID-19 and 5 patients with severe COVID-19 were visualized using uniform manifold approximation and projection (UMAP) for dimensionality reduction. The UMAPs of all samples and separate study groups are shown. (**D**) Median fluorescence intensity (MFI) heatmap of surface markers projected on UMAP. (**E**) Distribution of identified FlowSOM populations overlaid on the UMAPs. (**F**) Frequency of the FlowSOM populations within HC (*n* = 6) and patients with mild (*n* = 10) and severe (*n* = 5) COVID-19. (**G**) Heatmap of selected surface markers in the 8 FlowSOM populations (Pop). (**H**) The HyperFinder plugin in FlowJo was used to determine the shortest gating strategy for the identified FlowSOM populations. Fraction of mature-homeostatic (HC, *n* = 6; mild C-19, *n* = 10; sev C-19, *n* = 5), aged (HC, *n* = 6; mild C-19, *n* = 17; sev C-19, *n* = 6), immunosuppressive (HC, *n* = 6; mild C-19, *n* = 10; sev C-19, *n* = 8), immature non-degranulated (HC, *n* = 6; mild C-19, *n* = 10; sev C-19, *n* = 9), immature (HC, *n* = 6; mild C-19, *n* = 18; sev C-19, *n* = 8), immature-activated (HC, *n* = 6; mild C-19, *n* = 15; sev C-19, *n* = 7). Statistical analysis performed using 2-tailed Mann-Whitney test (**A**). Differences between HC and mild and severe groups were analyzed using ordinary 1-way ANOVA with Tukey’s multiple comparisons test. *P < 0.05; **P < 0.01. Median with IQR is shown.

We used flow cytometry to identify and characterize neutrophil populations in the whole blood of a subset of patients with COVID-19 and healthy controls. Total peripheral neutrophil count was increased with COVID-19 severity (Figure 1A). A manual gating strategy was used to define neutrophils as CD15^+^ or CD66b^+^ and CD14^–^ (Figure 1B). Dimension reduction using uniform manifold approximation and projection (UMAP) revealed that SARS-CoV-2 infection resulted in the emergence of discrete neutrophil phenotypes in patients with COVID-19 (Figure 1C). We then analyzed the expression of markers related to neutrophil activation, development, migration, and immunomodulation. To assess whether specific neutrophil populations were associated with disease severity, we based our initial analysis on these UMAP plots (Figure 1D), supplemented with flow self-organizing map (FlowSOM) clustering (Figure 1E). We defined 8 populations: populations 0 (67.9% of total dataset), 6 (14.3%), and 2 (8.6%) were the 3 main neutrophil populations in the combined dataset. These were accompanied by 5 rarer populations, populations 1 (2.65%), 5 (2.36%), 4 (2.04%), 3 (1.09%), and 7 (1.03%). However, there was a difference in the relative distribution of each patient group to each population (Figure 1F), suggesting that escalating COVID-19 severity leads to an overall change in marker expression by neutrophils. Population 1 (CD16^+^CD10^+^CD63^hi^CD62L^–^PD-L1^–^) was predominantly enriched in 1 healthy donor and virtually absent in others, whereas population 6 (CD16^+^CD10^+^CD62L^+^CXCR2^+^CXCR4^–^) (Figure 1, F and G), which contained CD16^+^CD10^+^ cells, was underrepresented in COVID-19. Notably, CXCR2 is a key chemokine receptor driving neutrophil migration and marks mature neutrophils in a steady state (25). Population 6 was characterized by high CXCR2 and low CXCR4 expression, suggesting a mature-homeostatic phenotype (Figure 1H). The mild COVID-19 group were overrepresented in population 0 (CD16^+^CD10^+^CD62L^+^CXCR2^+^CXCR4^+^) and population 7 (CD16^+^CD10^+^CD63^+^PD-L1^hi^LOX-1^+^CD62L^lo^). These 2 populations displayed high expression of CXCR4 and CD62L, suggestive of aged cells (25). Population 7 expressed PD-L1 and LOX-1, possibly indicating cells with an immunosuppressive phenotype (26, 27). Neutrophils from patients with mild and severe COVID-19 also contributed disproportionately to populations 2, 3, and 4, all of which shared a CD16^–^CD10^–^ phenotype, characteristic of immature neutrophils. Population 4 was CXCR2^lo^CD62L^–^, suggestive of an immature-activated phenotype. Population 5 (CXCR2^–^CD16^+^CD10^–^CD63^–^) was almost exclusively derived from patients with COVID-19 and displayed low CD63 expression, suggesting an immature non-degranulated phenotype. To enable identification of these populations in a wider patient cohort, we created an optimal gating hierarchy for each FlowSOM population informed by HyperFinder and further guided by fluorescence minus one (FMO) controls (Supplemental Figure 1). In this larger cohort, we verified a marked drop in population 6, mature-homeostatic neutrophils, in COVID-19; an increase in aged neutrophils (population 0) in mild COVID-19; and the emergence of immature non-degranulated neutrophils (population 5) in severe disease (Figure 1H). Individual marker expression by neutrophils was less able to discriminate healthy controls and patients with mild COVID-19 and severe COVID-19 (Supplemental Figure 2), though findings were consistent with the clustering-informed gating, with a clear stepwise increase in CD10^lo^ (Supplemental Figure 2A) and decrease in CXCR2 expression (Supplemental Figure 2B), respectively with COVID-19 disease severity. These findings highlight that COVID-19 progression is characterized by an accumulation of activated and immature neutrophils in patients with COVID-19. These findings suggest phenotypic dysregulation of the neutrophil compartment between COVID-19 patients with differences in disease severity.

### Severe COVID-19 is associated with a higher frequency of LDNs.

**Figure 2:**
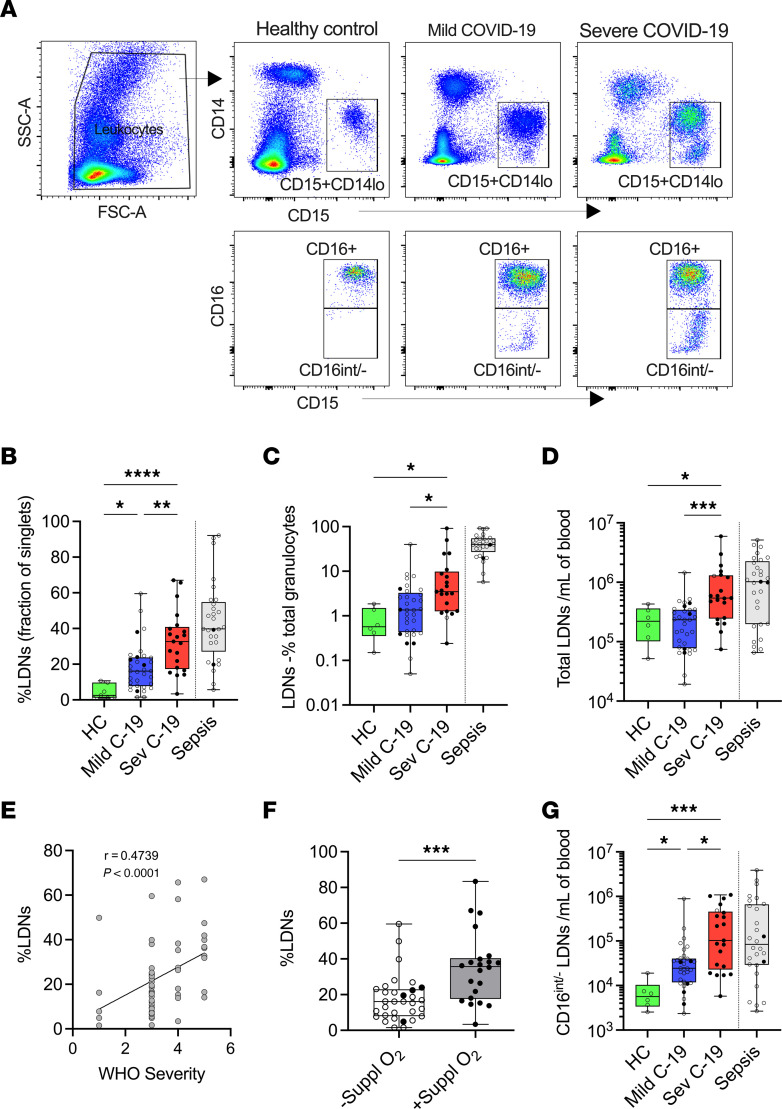
Severe COVID-19 is associated with a higher frequency of LDNs. (**A**) Gating strategy for identification of LDNs from PBMC fraction and discrimination of CD16^+^ and CD16^int/–^ subsets from different study groups. (**B**) Fraction of LDNs in PBMCs and (**C**) in total granulocytes and (**D**) absolute cell count from healthy controls (HC, *n* = 6) and patients with mild (*n* = 33) and severe (*n* = 21) COVID-19. (**E**) Spearman’s correlation between frequency of LDNs and WHO severity score (patients with score 1, *n* = 5; 3, *n* = 27; 4, *n* = 10; 5, *n* = 10). (**F**) LDN frequency in patients without (*n* = 32) or with (*n* = 22) oxygen supplementation. Statistical analysis was performed using Welch’s *t* test. ***P < 0.001. (**G**) Absolute cell count of CD16^int/–^ LDNs from HC (*n* = 6) and patients with mild (*n* = 33) and severe (*n* = 21) COVID-19. Solid black dots represent individuals with steroid exposure at the time of sampling. In panels **B**–**D** and **G**, the respective values in a contemporaneous cohort of patients with bacterial sepsis are shown in gray; these are not included in the statistical analysis and are shown for comparison purposes only. Differences between HC and mild and severe COVID-19 groups were analyzed using ordinary 1-way ANOVA with Tukey’s multiple comparisons test. *P < 0.05; **P < 0.01; ***P < 0.001; ****P < 0.0001. Median with IQR is shown.

We next asked if including cell density as a variable might improve the ability to discriminate between disease groups without the requirement to perform analysis of multiple phenotypic markers in combination. Thus, we analyzed CD15^+^CD66b^+^CD14^–^ neutrophils in the PBMC fractions from healthy controls and from patients with mild and severe COVID-19 (Figure 2A). The frequency of LDNs as a fraction of PBMCs increased progressively in patients with increasing COVID-19 severity to a level comparable with that observed in bacterial sepsis (Figure 2, B–D). Furthermore, the increase in LDN fraction in patients with COVID-19 was significantly associated with both WHO severity status (Figure 2E) and oxygen dependency (Figure 2F). Our results from whole blood neutrophil analysis suggested that the emergence of immature neutrophils is associated with severe COVID-19. Analysis of the LDN fraction further verified a marked expansion of CD16^int^ immature neutrophils with increasing severity of COVID-19 (Figure 2G).

### LDNs comprise phenotypically distinct subsets that are associated with severe COVID-19.

**Figure 3:**
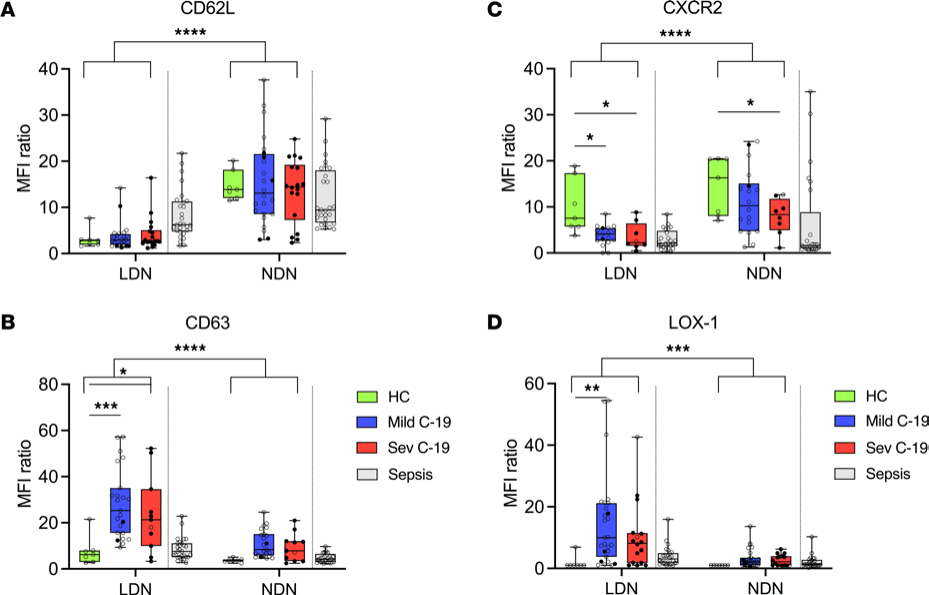
LDNs comprise phenotypically distinct subsets that are associated with severe COVID-19. PBMCs were isolated from fresh whole blood of controls (HC) and patients and surface stained for various neutrophil markers. MFI ratio of (**A**) CD62L (HC, *n* = 7; mild C-19, *n* = 24; sev C-19, *n* = 18), (**B**) CD63 (HC, *n* = 7; mild C-19, *n* = 23; sev C-19, *n* = 11), (**C**) CXCR2 (HC, *n* = 7; mild C-19, *n* = 18; sev C-19, *n* = 8), and (**D**) LOX-1 (HC, *n* = 7; mild C-19, *n* = 27; sev C-19, *n* = 16) expression on LDNs and NDNs. Solid black dots represent individuals with steroid exposure at the time of sampling. In each panel, the respective values in a contemporaneous cohort of patients with bacterial sepsis are shown in gray; these are not included in the statistical analysis and are shown for comparison purposes only. Statistical analysis was performed using 2-way ANOVA with Tukey’s multiple comparisons test. *P < 0.05; **P < 0.01; ***P < 0.001; ****P < 0.0001. Median with IQR is shown.

LDNs have been reported to display phenotypical and functional properties distinct from NDNs, which has implications for outcomes in multiple diseases (10, 15). We therefore sought to investigate whether LDNs or NDNs, or both, shared the aberrant phenotypic expression characteristics of whole blood neutrophils (Figure 1). Thus, we examined the differential expression of CD62L, CD63, CXCR2, and LOX-1 within each neutrophil fraction across the range of disease severity. We observed significantly downregulated CD62L expression in the LDN fraction but no alteration in this marker with COVID-19 disease severity (Figure 3A). On the other hand, CD63, a marker for azurophilic degranulation (28), was markedly upregulated in LDNs of patients with mild and severe COVID-19 (Figure 3B) (an effect not evident in whole blood, Supplemental Figure 2D). We also found strong disease-associated reduction of the chemokine receptor CXCR2 in the LDN fraction (Figure 3C). CXCR2 was the only marker downregulated in whole blood neutrophils of patients with both mild and severe COVID-19 (Supplemental Figure 2E). The immunosuppressive marker LOX-1 (27) was also differentially upregulated in patients with COVID-19, with the highest expression seen in mild COVID-19 (Figure 3D). Interestingly, while LDNs were abundant in patients with sepsis, the surface expression of the above markers on NDNs and LDNs differed from patients with COVID-19 (Figure 3, A–D).

**Figure 4:**
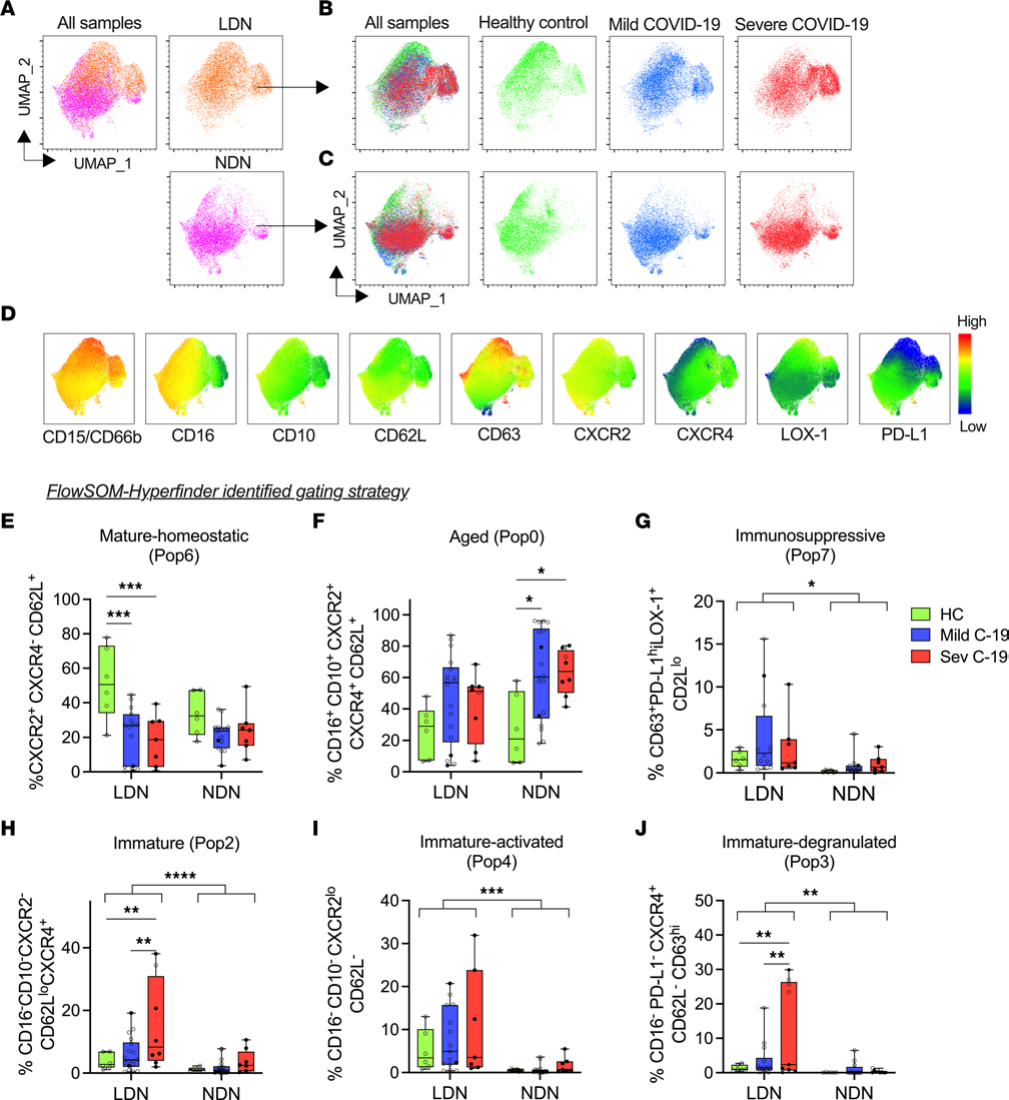
Whole blood–defined neutrophil phenotypes associated with severe disease are enriched in the LDN fraction. A total of 124,527 CD15^+^ neutrophils were concatenated from matched PBMCs and NDNs from healthy controls (HC, *n* = 6) and patients with mild (*n* = 10) and severe (*n* = 5) cases as in Figure 1. (**A**) UMAP of all samples and each fraction is shown. UMAP of healthy, mild, and severe groups within the (**B**) LDN and (**C**) NDN fractions. (**D**) MFI heatmap of selected surface markers projected on the UMAP shown in panel **A** (all samples). FlowSOM-HyperFinder–defined gating strategy was applied to matched LDN and NDN fractions from the same HC and patients with COVID-19. Fraction of (**E**) mature-homeostatic (HC, *n* = 6; mild C-19, *n* = 15; sev C-19, *n* = 7), (**F**) aged (HC, *n* = 6; mild C-19, *n* = 18; sev C-19, *n* = 8), (**G**) immunosuppressive (HC, *n* = 6; mild C-19, *n* = 12; sev C-19, *n* = 7), (**H**) immature (HC, *n* = 6; mild C-19, *n* = 18; sev C-19, *n* = 8), (**I**) immature-activated (HC, *n* = 6; mild C-19, *n* = 15; sev C-19, *n* = 7), and (**J**) immature-degranulated (HC, *n* = 6; mild C-14, *n* = 10; sev C-19, *n* = 9) neutrophil populations. Solid black dots represent individuals with steroid exposure at the time of sampling. Statistical analysis was performed using 2-way ANOVA with Tukey’s multiple comparisons test. *P < 0.05; **P < 0.01; ***P < 0.001; ****P < 0.0001. Median with IQR is shown.

UMAP analysis of paired LDNs and NDNs (Figure 4A) verified fraction-specific cluster patterns, supporting the substantial phenotypic differences between these 2 neutrophil fractions. When UMAPs of each neutrophil fraction were separated by study group (Figure 4, B and C), neutrophil clustering differed between patients with COVID-19 and healthy controls, with further differences in patients with mild or severe disease. Surface marker expression differed between the 2 neutrophil fractions projected onto the UMAP, with higher expression of markers such as CD15/CD66b, CD63, and LOX-1 and lower expression of CD62L, PD-L1, and CXCR2 observed in the LDN fraction (Figure 4D).

We next applied our FlowSOM-HyperFinder guided gating strategies, as initially defined on whole blood neutrophils (Figure 1G), to our LDN and NDN populations to investigate if this could further identify variables that were associated with disease severity. Mirroring the findings in whole blood, the mature-homeostatic immunotype was reduced in patients with COVID-19, in the LDN fraction (Figure 4E), while the proportion of aged neutrophils was increased primarily in NDNs (Figure 4F). Notably, the immunosuppressive PD-L1^hi^LOX-1^+^ neutrophil signal was almost entirely confined to the LDN subset, irrespective of disease state (Figure 4G). The immature neutrophil immunotypes of immature, immature-activated, and immature-degranulated were strongly enriched in the LDN fraction and significantly upregulated in patients with severe COVID-19 (Figure 4, H–J). The immature non-degranulated population 5 (CXCR2^–^CD16^+^CD10^–^CD63^–^) identified in whole blood in severe COVID-19 was too rare to accurately quantify in the LDN and NDN fractions. In summary, distinct neutrophil phenotypes emerged with escalating COVID-19 disease severity. Most of the divergent neutrophils related to immaturity, activation, and immunosuppressive features were confined to the LDN fraction.

### Neutrophils from patients with severe COVID-19 display impaired ROS and NET production.

**Figure 5:**
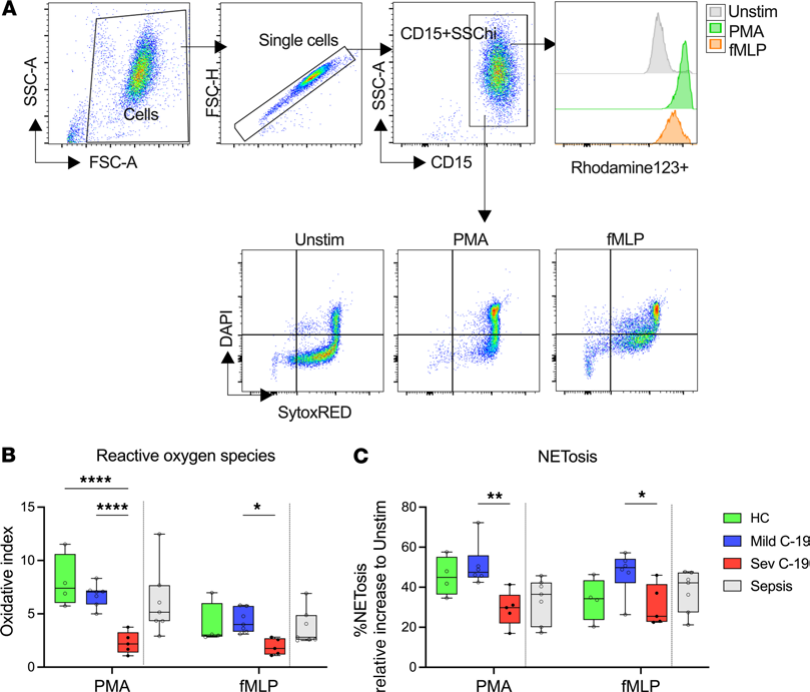
Neutrophils from patients with severe COVID-19 display impaired ROS and NET production. Fresh neutrophils were isolated from healthy controls and patients with COVID-19 and stimulated with PMA or fMLP to induce ROS and NET production. (**A**) Gating strategy to quantify NETs and ROS production in neutrophils. (**B**) Fold-increase in ROS production in stimulated NDNs over unstimulated NDNs isolated from healthy controls (HC, *n* = 4) and patients with mild (*n* = 7) and severe (*n* = 5) COVID-19. (**C**) Percentage NETosis in NDNs stimulated with PMA or fMLP from HC (*n* = 4) and patients with mild (*n* = 6) and severe (*n* = 5) COVID-19. Solid black dots represent individuals with steroid exposure at the time of sampling. In panels **B** and **C**, the respective values in a contemporaneous cohort of patients with bacterial sepsis are shown in gray; these are not included in the statistical analysis and are shown for comparison purposes only. Statistical analysis was performed using 2-way ANOVA with Tukey’s multiple comparisons test. *P < 0.05; **P < 0.01; ****P < 0.0001. Median with IQR is shown.

Production of reactive oxygen species (ROS) and neutrophil extracellular traps (NETosis) are 2 important functional roles that neutrophils play in inflammation (29). Given the clear alteration in neutrophil phenotype we observed in COVID-19, we hypothesized that this translated into altered neutrophil function, quantified by measurement of neutrophil ROS and NET production ex vivo following stimulation with PMA and N-Formylmethionine-leucyl-phenylalanine (fMLP). Neutrophils were identified as CD15^+^SSC^hi^ cells and analyzed to determine the oxidative index and fraction of SytoxRED^+^DAPI^+^ NETotic cells (Figure 5A). At basal level, we observed no difference in ROS or NET production between healthy controls and patients with mild or severe disease in either LDN or NDN fraction (Supplemental Figure 3, A and B). Surprisingly, although most COVID-19–associated neutrophil phenotypic changes were observed in the LDN fraction, all the functional associations with disease were in the NDN fraction. LDN ROS production and NETosis in response to broad or specific cell activation with PMA and fMLP, respectively, were similar between healthy controls and patients with COVID-19 (Supplemental Figure 3, C and D). In contrast, NDNs displayed markedly impaired function in severe disease, with a profound reduction in ROS production (Figure 5B). NETosis induced by the broad cellular activator PMA was also impaired in patients with severe COVID-19 (Figure 5C), whereas we observed enhanced NETosis in patients with mild COVID-19 when neutrophils were stimulated with the specific formyl peptide receptor activator fMLP (Figure 5C). Together these findings demonstrate that neutrophils in patients with severe COVID-19 are dysfunctional.

### Neutrophil arginase-1 expression remains elevated throughout the COVID-19 disease course through to convalescence.

**Figure 6:**
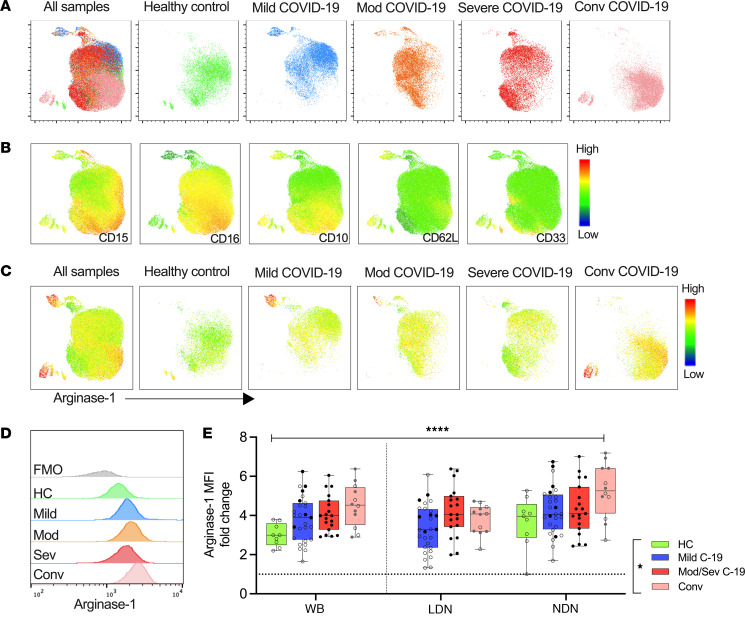
Neutrophil arginase-1 expression remains elevated throughout the COVID-19 disease course through to convalescence. Fresh whole blood was obtained from healthy controls, mild, moderate, severe, and convalescent patients and stained for basal intracellular arginase-1 expression. An equal number of neutrophils from each study group was concatenated and visualized using UMAP. (**A**) UMAP of all samples and separate study groups (healthy controls [HC], *n* = 3; mild, *n* = 4; moderate, *n* = 4; severe, *n* = 4; and convalescent, *n* = 5) is shown. (**B**) MFI heatmaps showing individual surface marker expression projected onto the UMAP. (**C**) MFI heatmaps showing intracellular expression of arginase-1 projected onto the UMAP and stratified by disease severity. (**D**) Overlaid histogram plot of intracellular arginase-1 expression in different disease groups. FMO control is shown for reference. (**E**) Intracellular arginase-1 expression was quantified in whole blood neutrophils and LDN and NDN fractions from HC (*n* = 9) and mild (*n* = 28) and moderate/severe (*n* = 19) COVID-19 patients and convalescent (*n* = 12) individuals. The MFI fold-change relative to FMO is presented. Solid black dots and gray dots represent individuals with steroid exposure at the time of sampling and during active disease, respectively. Statistical analysis was performed using mixed effects analysis with Tukey’s multiple comparisons test. *P < 0.05; ****P < 0.001. Median with IQR is shown.

Given the alteration in neutrophil function in COVID-19, we next investigated the impact on processing and release of arginase-1, a neutrophil-derived modulator of T cell function (30). To investigate the expression of arginase-1 by neutrophils throughout the disease course, additional whole blood samples from healthy controls, patients with COVID-19, and patients who had recovered from COVID-19 (convalescent) were stained for intracellular arginase-1. To assess the cellular distribution of arginase-1 within the neutrophil compartment, we generated UMAPs using whole blood neutrophils from 5 individuals in each group, which further verified that neutrophil phenotype changed with increasing COVID-19 severity and additionally showed that persistent changes remained evident in the convalescent period (Figure 6, A and B). We observed distinct populations of arginase-1–positive neutrophils from acute and, most notably, in convalescent COVID-19 patients (Figure 6C), which were also CD16^lo^CD10^int^CD62L^int^ (Figure 6B). Arginase-1 MFI was most elevated in convalescent samples (Figure 6D). We went on to quantify arginase-1 MFI fold-change against FMO in whole blood neutrophils, NDNs, and LDNs, in an expanded cohort (Figure 6E), which verified the increase in intracellular neutrophil arginase-1 expression in COVID-19 and most notably in convalescent individuals.

### COVID-19 neutrophils suppress T cell proliferation independent of arginase-1 and display an inability to release arginase-1.

**Figure 7:**
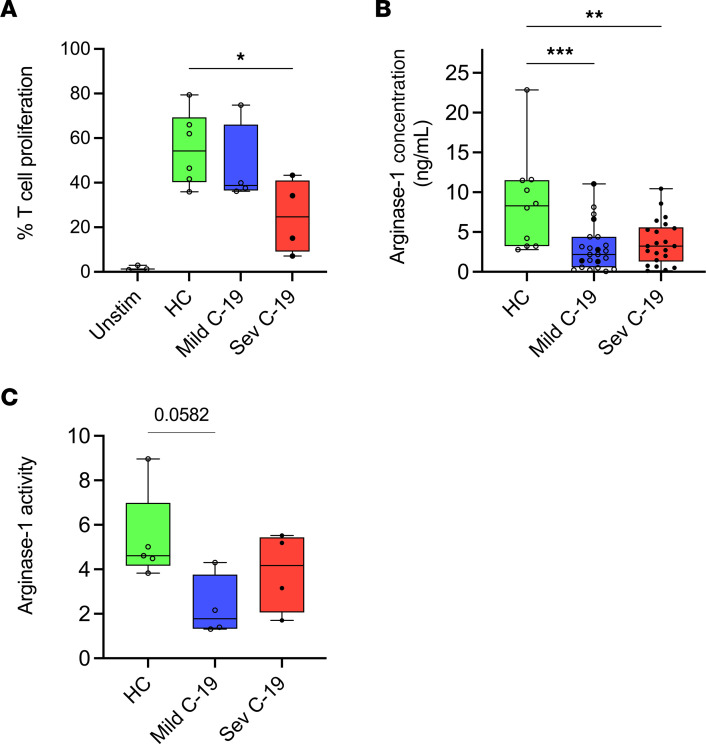
COVID-19 neutrophils suppress T cell proliferation independent of arginase-1 and display an inability to release arginase-1. PBMCs were isolated from autologous healthy controls and cocultured with cell-free supernatants prepared from isolated neutrophils cultured for 20 hours. (**A**) Rate of T cell proliferation upon coculture with neutrophil supernatant harvested from healthy controls (HC, *n* = 6) and patients with mild (*n* = 4) and severe (*n* = 6) COVID-19. (**B**) Arginase-1 concentration (determined by immunoassay) in the serum of HC (*n* = 10) and patients with mild (*n* = 23) and severe (*n* = 22) cases. (**C**) Arginase-1 activity in culture supernatants of purified neutrophils obtained from HC (*n* = 4) and patients with mild (*n* = 4) and severe (*n* = 4) cases. Solid black dots represent individuals with steroid exposure at the time of sampling. Statistical analysis was performed using Kruskal-Wallis test with Dunn’s multiple comparisons and ordinary 1-way ANOVA with Tukey’s multiple comparisons test. *P < 0.05; **P < 0.01; ***P < 0.001. Median with IQR is shown.

Arginase-1 is a potent immunosuppressive molecule for T cells (30). Given the increased arginase-1 expression, we went on to assess whether neutrophils from patients with COVID-19 possessed T cell–suppressive function by coculturing healthy anti-CD3ε– and anti-CD28–stimulated T cells with neutrophil supernatants. Compared with neutrophil-conditioned media form healthy controls, conditioned media from patients with severe COVID-19 suppressed the proliferation of activated T cells (Figure 7A). However, this suppression of T cell proliferation was not reversed by the addition of the arginase-1 inhibitor Nω-Hydroxy-nor-l-arginine monoacetate (nor-NOHA, Supplemental Figure 4A). Investigating this further, we found that serum arginase-1 concentrations were actually lower in both mild and severe COVID-19 (Figure 7B). Moreover, the neutrophil-conditioned media did not have elevated arginase enzyme activity compared to healthy controls (Figure 7C), and the degree of T cell proliferation did not correlate with either arginase-1 level or enzymatic activity (Supplemental Figure 4, B and C). Thus, neutrophils from patients with severe COVID-19 probably inhibit T cell activation by means other than release of arginase-1, and the cells appear to have an inability to effectively release arginase-1. To investigate further the factors driving arginase-1 expression in COVID-19, we built a generalized estimating equations (GEE) model (Table 2). This showed that CD62L expression, COVID-19 severity, and corticosteroid exposure were the strongest independent predictors in multivariate analysis, suggesting that neutrophil arginase-1 expression in COVID-19 is linked to cell activation and that the observations relating to arginase-1 expression in severe COVID-19 may be confounded by corticosteroid exposure.

### Dexamethasone does not alter intracellular arginase-1 expression but dampens arginase-1 release from healthy neutrophils.

**Figure 8:**
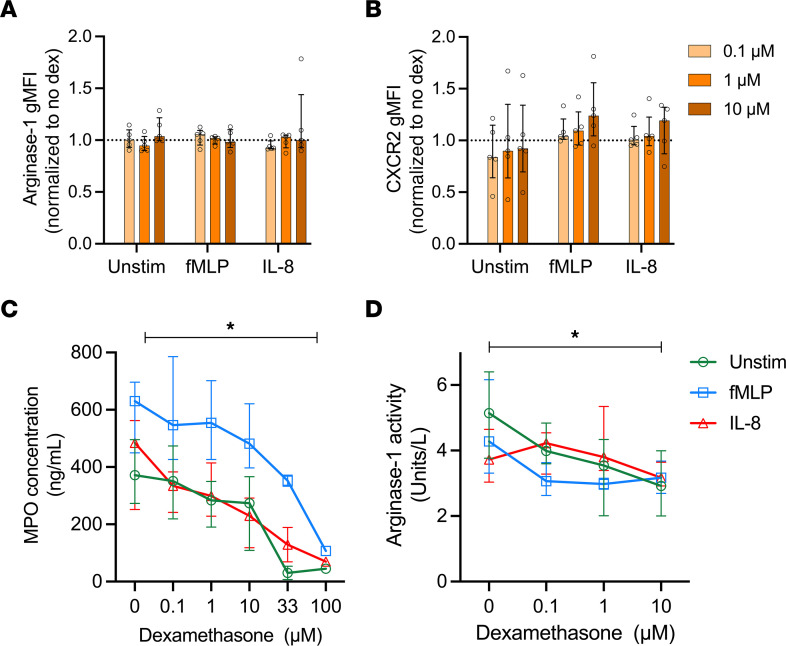
Dexamethasone does not alter intracellular arginase-1 expression but dampens arginase-1 release from healthy neutrophils. Fresh neutrophils were isolated from healthy controls and pretreated with varying concentrations of dexamethasone (0, 0.1 μM, 1 μM, 10 μM) for 4 hours. Cells were left unstimulated or stimulated with 5 μg/mL fMLP or 200 ng/mL IL-8. Geometric MFI (gMFI) ratio of (**A**) intracellular arginase-1 expression (*n* = 5) and (**B**) surface CXCR2 expression (*n* = 5) measured using traditional flow cytometry. (**C**) MPO release (*n* = 6; *n* = 2 with dexamethasone conc. 33 μM and 100 μM) and (**D**) arginase-1 activity (*n* = 6) measured in supernatants using ELISA and arginase-1 enzyme assay, respectively. Statistical analysis was performed using repeated measures 2-way ANOVA in **A**, **B**, and **D**. **C** was analyzed using mixed effects analysis. *P* values represent the effect of dexamethasone concentration, *P < 0.05. Median with IQR is shown.

To explore why neutrophils in patients with COVID-19 cannot release arginase-1 despite evidence of greater intracellular arginase-1 expression, we assessed the intracellular localization of this enzyme using imaging flow cytometry. We found that whole blood (WB) neutrophils obtained from a single patient with mild and a single patient with severe COVID-19 showed altered arginase-1 distribution in mild and severe COVID-19 (Supplemental Figure 5, A–C). In neutrophils from a single healthy control, arginase-1 staining was localized in discrete granules (Supplemental Figure 5A), whereas in the mild COVID-19 case there was diffused cytoplasmic staining (Supplemental Figure 5B). On the other hand, in a corticosteroid-exposed patient with severe COVID-19, there appeared to be a cytoplasmic staining pattern of arginase-1 (Supplemental Figure 5C) as well as an increase in granule count (Supplemental Figure 5, D–G). A recent study reported that dexamethasone treatment in patients with COVID-19 is associated with the appearance of neutrophil populations with increased *ARG1* gene expression (31). The promoter region of *ARG1* contains glucocorticoid response elements, suggesting that steroid exposure may affect arginase-1 expression (32, 33). Thus, considering the statistical association between corticosteroid exposure and arginase-1 expression and an apparent accumulation of intracellular arginase-1, we next sought to directly explore the effect of dexamethasone on neutrophils. We observed no increase in arginase-1 expression in healthy control neutrophils treated with dexamethasone (Figure 8A). Furthermore, there was no effect of dexamethasone treatment on intracellular expression pattern of arginase-1 (Supplemental Figure 6A) or on the number of arginase-1–stained granules following fMLP or IL-8 stimulation (Supplemental Figure 6B). We also measured the change in surface expression of CXCR2, the ligand for IL-8, to assess whether dexamethasone treatment may impact neutrophil migration. Dexamethasone treatment, at the highest concentration, led to a small but insignificant increase in CXCR2 expression in fMLP-stimulated and IL-8–stimulated neutrophils (Figure 8B). We next examined whether dexamethasone treatment affected neutrophil degranulation. Primary granules, which contain myeloperoxidase (MPO), are released last in the sequence of neutrophil activation. Pretreatment with dexamethasone modestly reduced the release of MPO from unstimulated neutrophils and those activated with fMLP or IL-8 in a dose-dependent manner (Figure 8C). While arginase-1, which is held mainly within the gelatinase granule compartment, was not induced in our hands by dexamethasone, we did find that release of active arginase-1 by neutrophils was also modestly inhibited by dexamethasone (Figure 8D). These findings suggest that, while dexamethasone may at least partially explain the observed reduction in release of arginase-1 from neutrophils, it cannot explain the increased expression of arginase-1 by neutrophils from patients with severe COVID-19 and those in convalescence.

## Discussion

Neutrophils can contribute to uncontrolled systemic inflammation in a variety of settings (34). A dysregulated immune response with pronounced neutrophilia is a feature of severe illness associated with COVID-19 (35). Our study aimed to extend our understanding of the impact of neutrophil phenotype and function on the clinical variability of COVID-19. Here, we report specific changes in the neutrophil compartment of patients with COVID-19 who develop severe disease. Central to our analysis was the ability to discriminate neutrophils based on density, showing that LDNs are a better proxy for disease severity than NDNs. Phenotypically, neutrophils from patients with COVID-19 displayed features associated with cellular activation, increased arginase-1 expression, and an increase in neutrophil subpopulations with immature and aged phenotypes. Additionally, we showed altered ex vivo function of neutrophils from patients with COVID-19.

We began our analysis by measuring the expression of individual surface markers on unfractionated WB neutrophils. We observed that certain markers (CXCR4, CXCR2, CD63, and LOX-1) showed a correlation with COVID-19 disease severity. Our analysis also revealed an increase in the frequency of CD10^int/–^ and CD16^int/–^ neutrophils, indicating that the presence of immature neutrophils in the blood may be due to emergency granulopoiesis. Using unsupervised clustering of flow cytometric data and automated gating algorithms on a subset of patients, we identified discrete neutrophil populations that we were then able to enumerate in our full dataset by retrospectively applying these gating structures. This approach led to the identification of cell populations that better distinguished neutrophils by disease severity. These gating strategies correlated with known neutrophil phenotypes. For example, we observed a decrease in the CXCR2^+^CXCR4^–^CD62L^+^ mature-homeostatic neutrophils and an increase in CD16^+^CD10^+^CXCR2^+^CXCR4^+^CD62L^+^ aged neutrophils in patients with COVID-19, indicating a potential link between these subpopulations and disease severity.

Our identification of distinct neutrophil phenotypes in patients with COVID-19 is consistent with prior studies that have reported heterogeneity in the circulating neutrophil pool of patients with varying COVID-19 disease severity (19, 36–40). We then examined the source of these neutrophil phenotypes by incorporating neutrophil density as a parameter. Here we found that most of the neutrophil phenotypes associated with severe disease were enriched in the LDN fraction. Indeed, LDN frequency itself was highly predictive of COVID-19 disease severity. Elevated LDN populations in patients with COVID-19 have been reported to correlate with disease severity (19, 20, 36, 41). Our study strengthens these findings and provides new insight for further comparison of neutrophil phenotypes in WB, granulocyte, and PBMC fractions. We found a much higher number of CD16^int/–^ immature neutrophils in the PBMC fraction than in WB. LDNs contain a higher proportion of immature neutrophils, even in healthy controls (42), but in COVID-19, both the proportion of LDNs with lower CD16 expression and the total number of these cells greatly increase over healthy individuals. By including neutrophil density as a metric, we found the predictive power of single-marker expression of CXCR2, CD63, and LOX-1 to be greatly enhanced, with the LDN fraction displaying the greatest phenotypic change with disease. Consistent with our findings, previous studies have reported decreased CXCR2 expression in neutrophils from patients with severe COVID-19 (19, 43). CXCR2 downregulation on neutrophils is implicated in the failure of neutrophil migration toward the infection loci in patients with severe sepsis (44–46). CXCR2 undergoes rapid internalization upon agonist stimulation (47). Since severe COVID-19 is associated with increased serum IL-8 concentrations (47–50), we hypothesize that this decreased CXCR2 expression could be a distal effect of excessive IL-8 being released by myeloid cells already recruited to the lungs, possibly suppressing further migration. Alternatively, the reverse migration of CXCR2^lo^ neutrophils from the inflamed lung to the peripheral blood might explain this phenomenon (51). The CD63^hi^ phenotype marks neutrophils that have undergone azurophilic degranulation (28), involving the release of MPO and neutrophil elastase, 2 proteins associated with NET formation. Patients with severe COVID-19 have higher gene (19, 43) and protein expression (37) of CD63, with this expression coinciding with neutrophil degranulation. LOX-1^+^ neutrophils, which are overrepresented in the LDN fraction, are thought to represent an immunosuppressive phenotype as seen in cancer (27). In COVID-19, other studies have reported an increased frequency of LOX-1^+^ neutrophils in severe cases (41, 43, 52). This highlights the value of LDNs in reflecting the neutrophil pathobiology in COVID-19. Our automated gating strategies also revealed greater changes within the LDN fraction. For example, the CD16^–^CD10^–^CXCR2^–^CD62L^lo^CXCR4^+^ cells (immature) displayed a robust increase in frequency with disease severity for LDNs but not NDNs. Conversely, there was a reduction in the mature homeostatic CXCR2^+^CXCR4^–^CD62L^+^ phenotype only in LDNs with disease severity. In contrast, the NDNs showed a greater increase in the CD16^+^CD10^+^CXCR2^+^CXCR4^+^CD62L^+^ aged phenotype.

Immature neutrophils characterized by low expression of CD16, CD10, and CXCR2 are now an accepted feature of severe COVID-19 disease (19, 39, 40, 43), with the increase in immature neutrophils being reported as a marker of poor prognosis (53). A subset of immature LDNs defined by CD123^+^LOX-1^+^ was elevated in patients with COVID-19 admitted to the intensive care unit versus non–intensive care unit (non-ICU) patients (54). Markers of neutrophil activation are among the most potent discriminators of critical illness in COVID-19. These markers are highly enriched in the developing neutrophil population, and it is proposed that neutrophil activation precedes the onset of critical illness and predicts mortality in COVID-19 (55). Since these developing neutrophils are enriched in the LDN fraction, these findings point to a potentially pathogenic role of these cells in driving COVID-19 disease.

We found that neutrophils from patients with severe, but not mild, COVID-19 displayed impaired ROS production. The respiratory burst is a core function of neutrophils that contributes to host defense (56). ROS release may be a contributing factor to lung injury leading to acute respiratory distress syndrome (ARDS) in COVID-19 (57). Consistent with our findings, others have reported impaired ROS production in neutrophils from patients with severe COVID-19 (19, 58). Neutrophils from ICU patients have higher basal ROS than non-ICU patients. However, upon stimulation, neutrophils from both ICU and non-ICU patients with COVID-19 fail to induce ROS production (59).

Patients with severe COVID-19 have NETs in their airways (3, 4, 8, 9), which has been suggested to be a key driver of immunothrombosis in COVID-19–induced ARDS (5–7, 9). Gene signatures associated with NETosis are enhanced in patients with severe COVID-19 (19, 40). However, we found reduced NETosis by neutrophils from patients with severe disease. Other groups have also shown that neutrophils from patients with severe COVID-19 fail to generate NETs in response to stimuli, though neutrophils from these patients may produce higher NETs at baseline (4, 8, 60). The literature on this topic is conflicting, as other groups have shown increased NETosis in patients with severe COVID-19. Masso-Silva et al. (61) show higher basal NET production in neutrophils from patients with COVID-19, which further increases in response to PMA in a dose-dependent manner. In our hands, we observed no difference in basal NET production between healthy controls and patients with COVID-19; thus, this difference could be due to an effect of sampling time or treatment status. LDNs have been shown to possess an enhanced ability to undergo NETosis as well as spontaneously form NETs (15, 16). Several of the NET-associated genes are upregulated in the LDN fraction of severe COVID-19 (19). In this study, we observed no change in NET formation in LDNs from patients with COVID-19 compared to healthy controls. We have demonstrated that unlike NDNs, which were phenotypically homogenous, LDNs contained diverse subpopulations. This difference could translate into their functional ability since the overall response of a homogenous NDN population would result in elevated ROS and NET production. Meanwhile, each LDN subpopulation may possess distinct functional responses, thus producing a net smaller response. Our findings contrast with the study by McLeish et al. (62), where LDNs from patients with severe COVID-19 underwent spontaneous and stimulated NET formation following bacterial stimulation. However, their study compared LDNs from patients with COVID-19 with NDNs from patients with COVID-19 and healthy controls, rather than LDNs from healthy controls. A limitation of our study is that we did not define the function of each of these neutrophil subsets that we identified using clustering and automatic gating but rather performed functional assays using total NDNs or LDNs.

We showed that supernatants produced by neutrophils from patients with severe COVID-19 had the capacity to reduce anti-CD3/28 mAb–induced T cell proliferation. Neutrophils can inhibit T cell proliferation in viral infections (26), and Cabrera et al. reported that NDNs from patients with severe COVID-19 inhibit T cell proliferation (41). Neutrophil populations with phenotypes like granulocytic–myeloid-derived suppressor cells (G-MDSCs) are reported in patients with severe COVID-19 (19, 63). G-MDSCs are defined by their ability to suppress immune responses by inhibiting T cell proliferation, promoting T cell anergy and recruiting regulatory T cells (64). One of the main mechanisms by which MDSCs suppress T cell responses is through alterations in l-arginine metabolism, most notably via arginase-1 expression (30). Surprisingly, we found that the neutrophil-conditioned media–induced suppression of T cell proliferation was independent of arginase-1. This is consistent with our discovery of an apparent block in the release of this protein from neutrophils from patients with severe COVID-19, leading to an accumulation of arginase-1–containing granules. In support of this, serum concentrations of arginase-1 were reduced in patients with COVID-19. IL-8–mediated neutrophil activation is required for degranulation of arginase-1 (65). We and others (43) have shown that CXCR2, the IL-8 receptor, is downregulated on neutrophils from patients with COVID-19. This may offer a mechanistic reason for the sequestration of arginase-1 inside neutrophils in patients with severe COVID-19, although we also demonstrated that dexamethasone reduces neutrophil degranulation of both MPO and arginase-1 directly. Dexamethasone therapy is frequently used in the treatment for COVID-19, and Sinha et al. showed that this treatment results in emergence of immature neutrophil populations with increased *ARG1* gene expression (31). Thus, our finding of increased intracellular arginase-1 with escalating COVID-19 severity is likely driven both by the infection itself and by the exogenously administered corticosteroids. However, we did not find increased arginase-1 protein in dexamethasone-treated neutrophils, suggesting that this effect may be targeting developing neutrophils in the bone marrow. Additionally, none of the recruits with convalescent COVID-19 had received corticosteroids for at least 6 weeks but had markedly increased arginase-1 levels, indicating that there are COVID-19–specific factors at play over and above the corticosteroid effect.

Our finding of enhanced intracellular arginase-1 expression further in convalescence contrasts with Dean et al., who report decreased arginase-1 protein levels in neutrophil lysates in convalescent individuals compared with patients with severe COVID-19 (66). These observed differences could be due to our detection by flow cytometry of arginase-1 expression within neutrophils opposed to Dean and colleagues’ quantification of arginase protein in cell lysates. Further, the sampling time after infection may not be the same; convalescent individuals in our cohort were sampled at a median of 78 days after infection, while the patient sampling interval is not indicated in the former study. The markedly elevated arginase-1 level in the convalescent period is remarkable given the short half-life of neutrophils and indicates a persisting transcriptional drive in the bone marrow long after the patient has recovered. Epigenetic effects in myeloid precursors may account for this, similar to the central trained immunity observed in monocytes after vaccination (67).

In conclusion, we have dissected the alterations in neutrophil phenotype and function in COVID-19, many of which are observed in the LDN cell fraction. Critically, we identify a hitherto-unreported block in arginase-1 release in severe COVID-19. Determining the relative contribution of SARS-CoV-2 infection and corticosteroid treatment to this observation will be challenging given the current standard approach to treating severe COVID-19 with this agent such that it will be impossible to separate these 2 drivers in the translational medicine setting. Newer approaches to studying this infection using animal or 3D culture models may provide additional insights to identify therapeutic targets in the neutrophil architecture.

## Methods

### Sex as a biological variable.

Our study involved both male and female participants. We found no significant differences in our results when sex was considered a biological variable.

### Patient recruitment.

Patients with active and convalescent COVID-19, confirmed by PCR, were recruited as part of the STTAR Collaboration Bioresource for COVID-19 (68) between September 2020 and January 2022. The dominant SARS-CoV-2 strains circulating during this period were Alpha and Delta. Peripheral blood was collected in lithium heparin tubes on the day of recruitment. Clinical data, including corticosteroid exposure, were obtained from the STTAR Bioresource. Age- and sex-matched healthy controls were recruited from the Rare Kidney Disease Registry and Biobank (69) and STTAR Bioresource. Patients with bacterial sepsis were used as an external comparator and were recruited from Tallaght University Hospital between February 2021 and July 2021, and between January 2023 and July 2023. Sepsis was defined as suspected or documented infection and an acute increase of 2 points on the Sequential Organ Failure Assessment scale (70).

### PBMC and neutrophil isolation.

Venous blood samples were collected in lithium-heparin vacutainers (BD). PBMCs and neutrophils were isolated by a modified Percoll (GE Healthcare, now Cytiva) gradient centrifugation method. Briefly, an equal volume of 2% dextran (MilliporeSigma) was added to 6–12 mL blood and inverted 20 times. Erythrocytes were left to sediment by gravity for 30 minutes; the supernatant was then spun at 200*g* with no brake. The pellet was resuspended in 3 mL of 55% Percoll, slowly layered over 4.5 mL of 65% Percoll, and spun with no brake on for 30 minutes at 1,500*g*. The PBMC and neutrophil layers were carefully removed into fresh Falcon tubes (Corning) and washed with PBS. Cells were then resuspended in 1 mL of FACS buffer (2% FBS in PBS). Viability was determined using Trypan blue (Gibco) and in all cases was found to be more than 90%. To create a neutrophil-conditioned supernatant, we isolated neutrophils from 6 healthy controls and 4 mildly and 4 severely ill patients and incubated the isolated neutrophils in complete RPMI at 37°C, 5% CO_2_, for 20 hours. Cell-free supernatant was harvested after centrifugation and stored at –80°C.

### Flow cytometry.

Isolated PBMCs, neutrophils, and WB were stained immediately for surface markers. Arm-to-stain time was less than 4 hours in all cases. A total of 1 × 10^6^ to 2 × 10^6^ PBMCs or neutrophils or 100 μL WB was surface stained for 20 minutes at room temperature (RT) in the dark with the fluorescently labeled anti-human mouse monoclonal antibody cocktail containing CD66b-PerCP-Cy5.5 (BioLegend, clone G10F5), CD15-PerCp-Cy5.5 (BioLegend, clone W6D3), CD14-PE-Cy7 (Thermo Fisher Scientific, clone 61D3), CD16-APC-Cy7 (BioLegend, clone 3G8), CD10-BV421 (BioLegend, clone HI10a), CD62L-BV510 (BioLegend, clone DREG-56), CD63-BV605 (BioLegend, clone H5C6), CXCR2-PE-Cy5 (BioLegend, clone 6C6), CXCR4-PE (BioLegend, clone 12G5), LOX-1–PE-Vio615 (Miltenyi Biotec, clone REA1188), PD-L1–BV650 (BD Biosciences, clone MIH1), CD3-FITC (BioLegend, clone HIT3a), CD19-FITC (BioLegend, clone HIB19), and CD56-FITC (Thermo Fisher Scientific, clone TULY56). Cells were then fixed and red blood cells lysed using 1× BD FACS lyse solution and incubated for 10 minutes at RT in the dark. Cells were washed thrice by centrifuging at 400*g* for 5 minutes, following which the cell pellets were resuspended in 200 μL of FACS buffer (2% fetal calf serum in PBS) and stored at 4°C until acquisition. FMO controls were prepared for each marker and used to define positive staining. Additionally, single stained controls to compute compensation were prepared using OneComp beads (eBioscience). Samples were acquired using the 3-laser LSRFortessa (BD Biosciences) using the FACSDiva version 8.0.1 (BD) software. Data were analyzed using the Kaluza software version 2.1 (Beckman Coulter) and FlowJo software v10.0.7 (BD). Cell debris and doublets were excluded, and neutrophils in each layer were defined as CD15^+^/CD66b^+^CD14^–^ cells.

Flow cytometric data were normalized using CytoNorm (v0.0.7) in R Studio v4.0.5 before concatenation in FlowJo. To carry out an unsupervised phenotypic analysis of neutrophil subsets, we concatenated 126,000 neutrophils (3,000 cells/sample) from PBMCs and isolated neutrophils from 6 healthy controls and 10 patients with mild and 6 patients with severe COVID-19 as described in Figure 1. Neutrophils within each group were downsampled to an equal number of cells for uniform visualization. The concatenated data were subjected to unsupervised dimensionality reduction and identification of cell populations by UMAP for dimension reduction v3.1 (71). Unbiased cell populations were identified using the FlowSOM (v3.0.18) clustering approach (72). The ideal gating strategy to identify the FlowSOM-guided population was then defined using HyperFinder v0.6.8.

### Intracellular ROS and NETosis quantification.

ROS and NET production in neutrophils was measured using a modified protocol combining the dihydrorhodamine123 (DHR123) assay to measure ROS and the DNA-dye-based NETosis assay developed by Zharkova et al. (73). Neutrophils were resuspended at 2 × 10^6^/mL in RPMI (Gibco) media containing 0.5% BSA. Cells were loaded with DHR123 (2.5 μg/mL) and Cytochalasin B (5 μg/mL) and incubated at 37°C, 5% CO_2_, for 15 minutes. We added 200,000 cells/100 μL to each well in a round-bottom, 96-well plate. Cells were primed with recombinant human TNF-α (2 ng/mL, Thermo Fisher Scientific) for 30 minutes at 37°C, 5% CO_2_, prior to stimulation. After priming, the cells were left unstimulated or stimulated with 100 μL of PMA (100 ng/mL, MilliporeSigma) or fMLP (5 μg/mL, MilliporeSigma), for 1 hour at 37°C, 5% CO_2_. After stimulation, 100 μL of 4% paraformaldehyde (PFA; final concentration 1.3%) was added to the cells and incubated in the dark for 15 minutes. Cells were washed at 100*g* for 10 minutes, and the pellet was resuspended in FACS buffer and stored overnight at 4°C. The next day, cells were stained with an anti–CD15-PerCP-Cy5.5 (clone W6D3) antibody. Cells were then washed twice with PBS and resuspended in 200 μL of DNA dye master mix containing DAPI (0.3 nmol/mL, Thermo Fisher Scientific) and SytoxRED (5 nmol/mL, Thermo Fisher Scientific) made up in FACS buffer and incubated for 15–20 minutes. The cells were immediately acquired on the flow cytometer. Intracellular ROS was quantified by measuring the fold-change in MFI of DHR123 between stimulated and unstimulated neutrophils. The rate of NETosis was quantified as the fraction of SytoxRED^+^DAPI^+^ neutrophils.

### Intracellular arginase-1.

PBMCs, NDNs, and WB were surface stained with the following anti-human monoclonal antibodies: CD15-PerCp-Cy5.5 (clone W6D3), CD14-PE-Cy7 (clone 61D3), CD16-APC-Cy7 (clone 3G8), CD10-BV421 (clone HI10a), CD62L-FITC (clone DREG-56), and CD33-APC (clone WM53) or CXCR2-APC (clone 6C6) followed by fixation and RBC lysis using 1× FACS lysing solution. Cells were then permeabilized with 1 mL of 0.2% saponin in FACS buffer for 10 minutes, 2 mL of 20% blocking buffer was added for another 10 minutes, and the cells were washed at 400*g* for 5 minutes. After discarding the supernatant, 50 μL was left in the tube, and 50 μL of 0.2% saponin and 3 μL of anti-human arginase-1 (BioLegend, clone 14D2C43) were added to the cells and incubated for 15 minutes in the dark at RT. Cells were then washed with PBS and resuspended in 200 μL of 2% PFA and stored overnight. Cells were then centrifuged for 10 minutes in 2% PFA and resuspended in FACS buffer to ensure the integrity of fluorophores. Cells were acquired on the BD FACSCanto II. For imaging flow cytometry, 1 million stained WB, PBMCs, and NDNs were incubated with 0.2 μg/mL DAPI (MilliporeSigma) for nuclear staining before acquisition on an ImageStream X MkII imaging flow cytometer (Cytek biosciences) using INSPIRE data acquisition software (Cytek biosciences). Compensation and data analysis were performed using IDEAS 5.0 software (Cytek biosciences).

### T cell suppression assay.

PBMCs from healthy controls were resuspended at 2 × 10^6^ cells/mL in RPMI (Gibco) containing 10% FBS and incubated with CellTrace Violet (CTV) (2.5 mM, Thermo Fisher Scientific) for 15 minutes. We added 200 μL of neutrophil-conditioned supernatant to 96-well plates coated with 5 μg/mL of anti-CD3, followed by 100 μL of CTV^+^ or CTV^–^ PBMCs and 5 μg/mL of anti-CD28. We added 40 ng/mL of the arginase-1 inhibitor nor-NOHA (EMD Millipore) to selected wells and monitored T cell proliferation. After 4 days, cells were centrifuged for 3 minutes at 400*g* and washed with PBS. LIVE/DEAD Fixable Aqua Dead Cell Stain Kit (Thermo Fisher Scientific) and FcX (Fc receptor blocking reagent) were added to cells and incubated for 30 minutes at 4°C. A total of 50 μL of antibody cocktail containing anti-CD3 APC-Cy7 (clone OKT3, BioLegend), anti-CD8 BV711 (clone SK1, BioLegend), and anti-CD4 BV605 (clone SK3, BioLegend) antibodies was prepared and added to each well, except live/dead, unstained, and CTV-only wells, and incubated for 20 minutes in the dark at RT. Cells were washed with FACS buffer, centrifuged at 400*g* for 3 minutes, and fixed with 50 μL IC fixation buffer (Thermo Fisher Scientific) for 10 minutes before acquisition.

### Arginase-1 serum ELISA.

The arginase-1 ELISA was carried out on serum samples from healthy controls and patients with COVID-19 as per the manufacturer manual (HK386-02, Hycult Biotech). Briefly, 100 μL in duplicate of standard, samples, and controls was added into appropriate wells. Samples were diluted 1:2 with dilution buffer. The plate was covered and incubated for 1 hour at RT, after which it was washed 4 times with wash buffer. A total of 100 μL of diluted tracer was added to each well, covered, and incubated for 1 hour at RT. A total of 100 μL of diluted streptavidin-horseradish peroxidase (strep-HRP) was added to each well and incubated for 1 hour at RT. A total of 100 μL of TMB substrate was added to each well. The plate was covered with aluminum foil and incubated for 30 minutes at RT before being quantified at 450 nm in a plate reader.

### Arginase-1 activity assay.

The arginase-1 activity assay was conducted as per the manufacturer manual (MilliporeSigma). A total of 20 μL of neutrophil-conditioned media from healthy control and patients with COVID-19 was added to a well in a flat-bottom, 96-well plate; 20 μL of recombinant arginase-1 at 1 μg/mL, 100 ng/mL, and 10 ng/mL were added to separate wells. Next, 25 μL of prepared 1 mM urea standard and 25 μL of water as a blank were added to separate wells. We added 5 μL of 5× substrate buffer to each sample. Substrate buffer was prepared using 4:1 of arginine buffer and manganese solution, respectively. The plate was covered and incubated for 2 hours at 37°C. The reaction was stopped by adding 80 μL of urea reagent to each well. Then 5 μL of 5× substrate buffer was added to the sample blank wells, followed by 1-hour incubation at RT. The absorbance was measured at 450 nm.

### Dexamethasone treatment.

Isolated neutrophils were resuspended at 2 × 10^6^ cells/mL in RPMI (10% FCS). Cells were treated with 0.1 μM, 1 μM, or 10 μM dexamethasone (Tokyo chemicals) solution and incubated for 4 hours at 37°C, 5% CO_2_. After incubation, 1 mL of cold PBS was added to the cells to stop the treatment, and the cells were centrifuged at 400*g* for 5 minutes. The supernatant was discarded, and the cell pellet was resuspended in RPMI media (10% FCS). Cells were then treated with 5 μg/mL of cytochalasin B for 15 minutes and subsequently left unstimulated or stimulated with 5 μg/mL of fMLP, or 200 ng/mL of recombinant IL-8, for 1 hour at 37°C, 5% CO_2_. The reaction was stopped by keeping the plate on ice for 2 minutes, and the cells were spun at 400*g* for 5 minutes. Cells were stained for intracellular arginase-1 analysis using either imaging or traditional flow cytometry as described in the previous sections. The supernatant was aliquoted and stored at –20°C.

### MPO ELISA.

MPO release was measured using a commercially available ELISA kit (R&D Systems, Bio-Techne) according to the manufacturer’s instructions. Briefly, high-binding, 96-well ELISA plates were coated with the capture antibody and incubated overnight at RT. Plates were washed with PBS containing 0.05% Tween and blocked by adding reagent diluent (1% BSA in PBS) and left to incubate at room temperature for 2 hours. Serial dilutions of the MPO standard were performed to achieve a concentration gradient of 4,000 pg/mL to 62.5 pg/mL. Samples were diluted 1:400 using the reagent diluent. After blocking, the plates were washed, and the standards/samples were added to appropriate wells and incubated for 2 hours at RT. Plates were washed again, followed by incubation with the detection antibody. Subsequently, strep-HRP was added to each well and allowed to incubate for 20 minutes. Next, detection antibody was washed off and substrate solution was added to each well and further incubated for 30 minutes, after which stop solution (1 M H_2_SO_4_) was added to halt color development. Absorbance was measured at 450 nm using an ELISA plate reader.

### Statistics.

Prism version 9 (GraphPad) was used to produce graphs. Statistical significance was calculated by performing 1-way or 2-way ANOVA with post hoc assessment or paired or unpaired 2-tailed *t* test as indicated. Correlation coefficients were generated using Spearman’s correlation analysis. Data are presented as median with IQR. A *P* value less than 0.05 was considered significant. The number of individuals within each group is indicated in the figure legends. We investigated the factors associated with arginase-1 levels in LDNs and NDNs. To simultaneously model both neutrophil fractions, we used a GEE model. Univariate GEEs were first fitted to evaluate the effect of risk factors on the response separately. Subsequently, a multivariate GEE was deployed to specify a correlation structure for arginase-1 in the layers. For data analysis, R (version 4.2.2) and Stata (version StatSE17) were used.

### Study approval.

The study was approved by institutional ethics committees of St James’s Hospital/Tallaght University Hospital, and all recruits provided written informed consent.

### Data availability.

Supporting data, including values for all data points shown in graphs and mean values, are available in the Supporting Data Values file.

## Author contributions

AD and AUM conducted experiments, performed formal data analysis, and wrote the original draft of the manuscript. AD conducted additional experiments and edited the final drafts of the manuscript, which were used to determine the authorship order. MC, IB, RSB, and CMF performed additional experiments and analyses. BK conducted regression analysis. SHP, LO, AMG, and JS collected clinical data. EN and SM recruited patients with sepsis and collected sepsis clinical data. HCH, PGF, NC, CNC, and MAL performed clinical data analysis. MAL and CMF supervised the study, provided training, and wrote the manuscript. MAL conceptualized the study and provided funding. All authors reviewed and approved the manuscript.

## Supplementary Material

Supplemental data

Supporting data values

## Figures and Tables

**Table 1:**
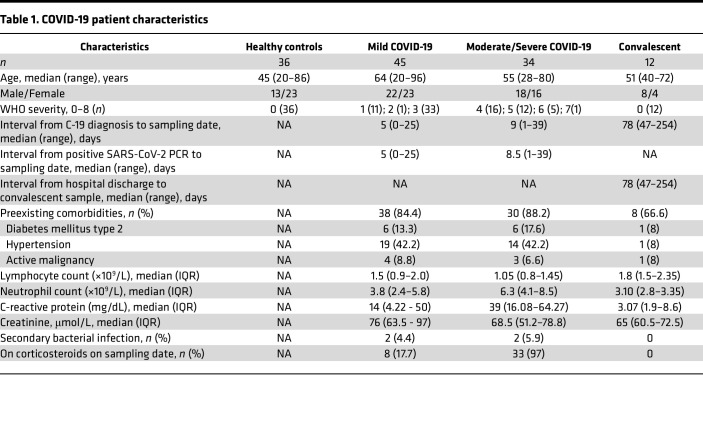
COVID-19 patient characteristics

**Table 2:**
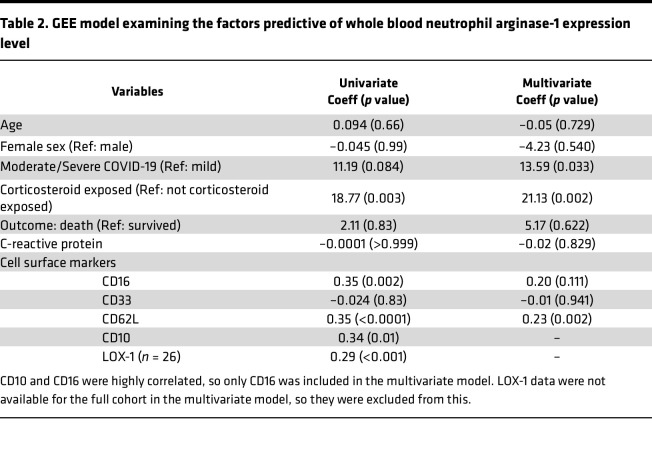
GEE model examining the factors predictive of whole blood neutrophil arginase-1 expression level

## Version 1 09/10/2024

Electronic publication
