# Supplementary material for: Emergence of dysfunctional neutrophils with a defect in arginase-1 release in severe COVID-19
Source: JCI Insight. 2024 Sep 10;9(17):e171659. doi: 10.1172/jci.insight.171659 (PMC11385094; doi:10.1172/jci.insight.171659)
Supplement: Supplemental data [file jciinsight-9-171659-s027.pdf]

STTAR COVID Bioresource members

William McCormack[1], Nicole Wood[1], Aideen Long[1], Orla Shiels[1], Padraic G. Fallon[1], Anne Marie McLaughlin[1], Ross McManus[1] , Mark Little[1], Liam Townsend[1,2], Colm Bergin[1,2], Adam Dyer[1,3], Martina Hennessy[1,3], Deirdre Reidy[3], Cliona Ní Cheallaigh[1-3] , Ignacio Martin Loeches[1,4] , Ana Rakovac[1,5], Seamas Donnelly[1,6] and Niall Conlon[1-3, 7]

Affiliations:

[1] Department of Clinical Medicine, School of Medicine, Trinity Translational Medicine Institute, Trinity College Dublin, Dublin, Ireland

[2]Department of Infectious Diseases, St. James's Hospital, Dublin, Dublin, Ireland

[3] Clinical Research Facility, St. James's Hospital, Dublin, Dublin, Ireland

[4] Department of Intensive Care Medicine, St James's Hospital, Dublin, Ireland

[5] Departments of Clinical Chemistry and Laboratory Medicine, Dublin 24 and School of Medicine, Tallaght University Hospital, Trinity College Dublin, Dublin, Ireland

[6] Department of Respiratory Medicine, Tallaght University Hospital, Dublin, Ireland

[7] Department of Immunology, St James's Hospital, Dublin, Ireland

Contents

Supplementary Table 1: Clinical characteristics of sepsis patient 2

Figure S1: FlowSOM-HyperFinder defined automated gating strategy.....................................3

Figure S2: Single marker phenotypic characterisation of whole blood neutrophils from COVID-19 patients...4

Figure S3: Assessment of ROS and NET production in LDN from COVID-19 patients. 5

Figure S4: Neutrophil mediated T-cell suppression in severe patients is arginase-1 independent. 6

Figure S5. Imaging flow cytometry suggests aberrant intracellular arginase-1 expression in COVID-19 neutrophils. 7

Figure S6. Dexamethasone treatment has no effect on intracellular arginase-1 expression pattern in healthy neutrophils. 8

Characteristics	Sepsis
**Participants*, n***	29
**Age, median (range), years**	63 (24-82)
**Male, *n (%)***	16 (55)
**SOFA Score, median (IQR)**	7 (5-8.5)
**Shock, *n (%)***	8 (28)
**Disease duration at sampling, median(range), days**	1 (0-11)
**Lymphocyte count ($x10^9$/L), median (IQR)**	1.1 (0.45-1.7)
**Neutrophil count ($x10^9$/L), median (IQR)**	11.5 (6.5-17)
**CRP (mg/dL), median (IQR)**	188 (121-350)
**Creatinine (µmol/L), median (IQR)**	101 (69-144)
**Causative organism, *n (%)***	
Gram negative bacilli	10 (35)
Gram positive cocci	12 (42)
Fungi	7 (24)
Unknown	10 (35)

Supplementary Table 1: Clinical characteristics of sepsis patient

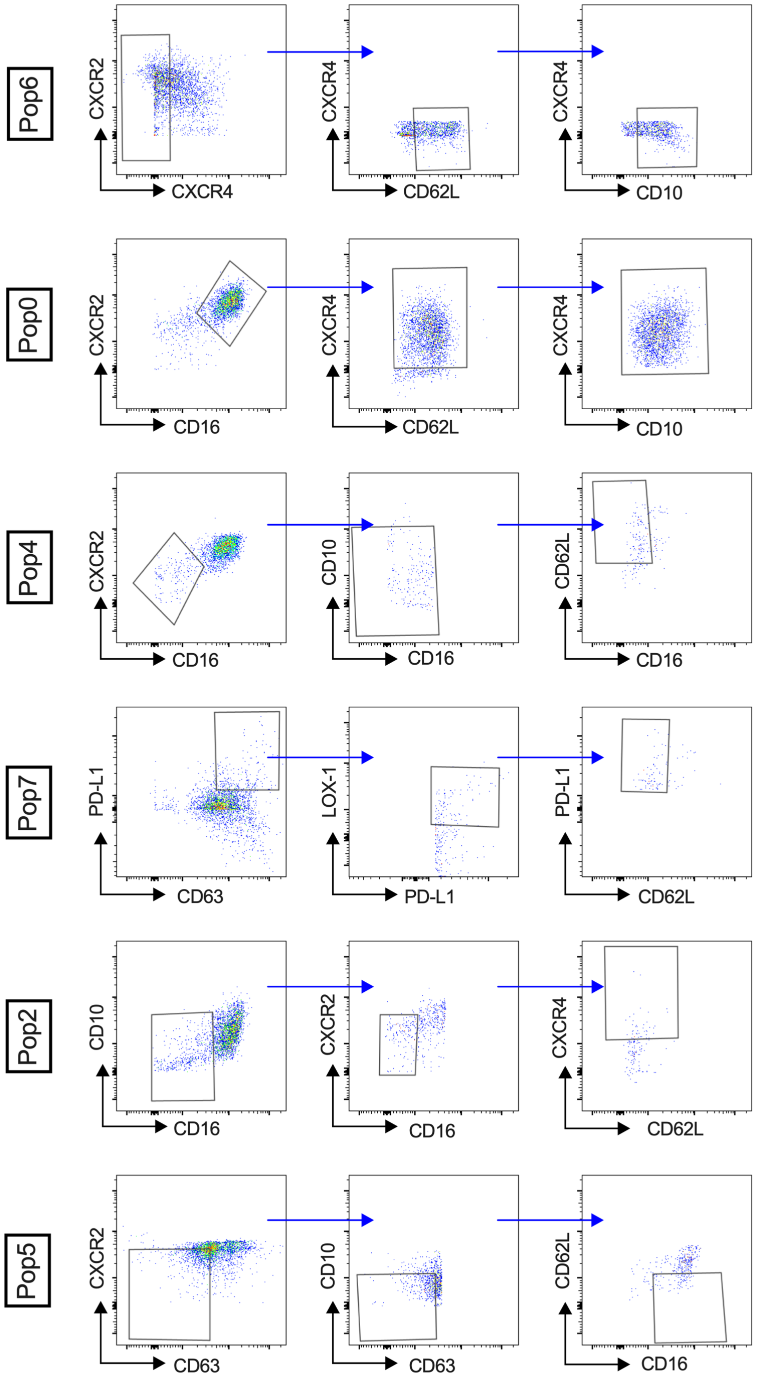

Figure S1: FlowSOM-HyperFinder defined automated gating strategy

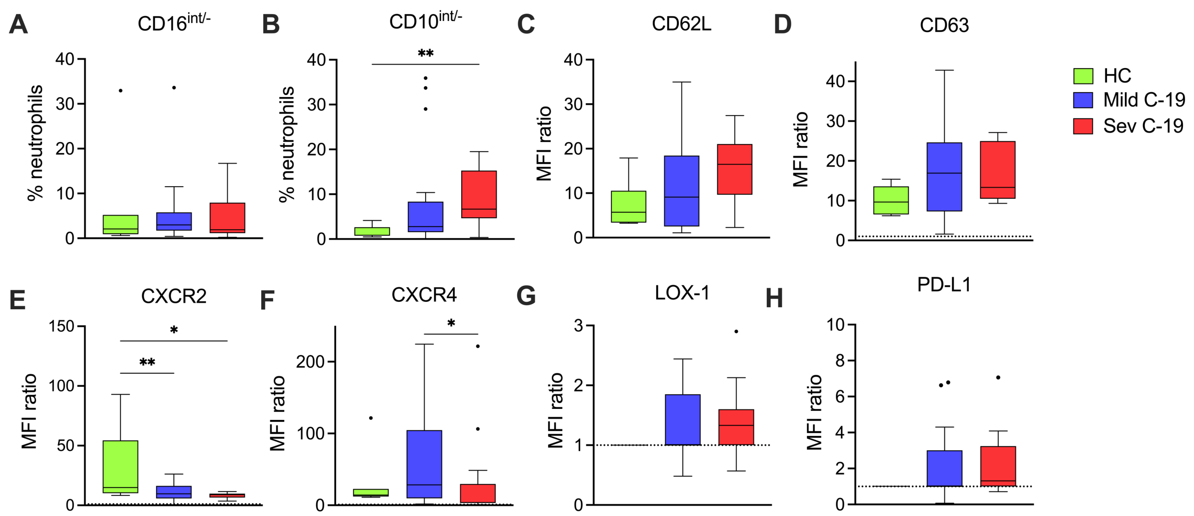

Figure S2: Single marker phenotypic characterisation of whole blood neutrophils from COVID-19 patients. 100ul of fresh whole blood obtained from patients with COVID-19 and healthy control was stained with surface markers for neutrophils, following which RBCs were lysed. **(A)** Fraction of CD16int/- and **(B)** CD10- neutrophils from HC (n= 7), patients with mild COVID-19 (n= 31) and severe COVID-19 (n= 17). MFI ratio of surface expression of **(C)** CD62L (HC, n=7; Mild C-19, n=24; Sev C-19, n=17), **(D)** CD63 (HC, n=7; Mild C-19, n=24; Sev C-19, n=10), **(E)** CXCR2 (HC, n=7; Mild C-19, n=18; Sev C-19, n=7), **(F)** CXCR4 (HC, n=7; Mild C-19, n=24; Sev C-19, n=17), **(G)** LOX-1 (HC, n=7; Mild C-19, n=27; Sev C-19, n=15), and **(H)** PD-L1 (HC, n=7; Mild C-19, n=31; Sev C-19, n=18). Statistical analysis was performed using Kruskal-Wallist test with Dunn's multiple comparisons test (B) and One-way ANOVA with Tukey's multiple comparison's test (E). Box and whiskers represent median with Tukey method (individual points are outliers). *p<0.05; **p<0.01.

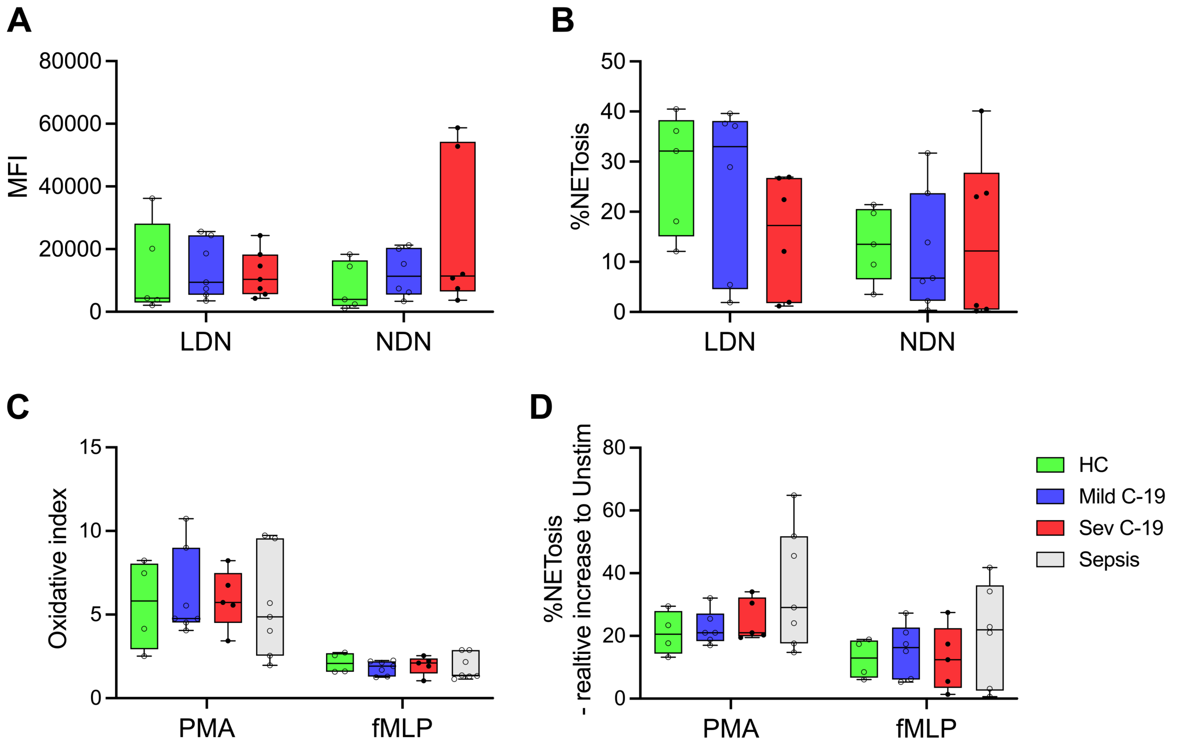

Figure S3: Assessment of ROS and NET production in LDN from COVID-19 patients.

Fresh neutrophils were isolated from healthy controls and patients with COVID-19 and stimulated with PMA or fMLP to induce ROS and NET production**. (A)** Basal ROS MFI and **(B)** Basal % NETosis in LDN and NDN from healthy controls (HC, n=5), mild (n=6) and severe (n=6) patients with COVID-19. **(C)** Fold increase in ROS production in stimulated LDN over unstimulated LDN (HC, n=4; mild, n=7 and sev C19, n=5), **(D)** Percent increase in NETosis in stimulated LDN (HC, n=4; mild, n=6 and sev C-19, n=5) from healthy controls and patients with COVID-19. Median with IQR is shown.

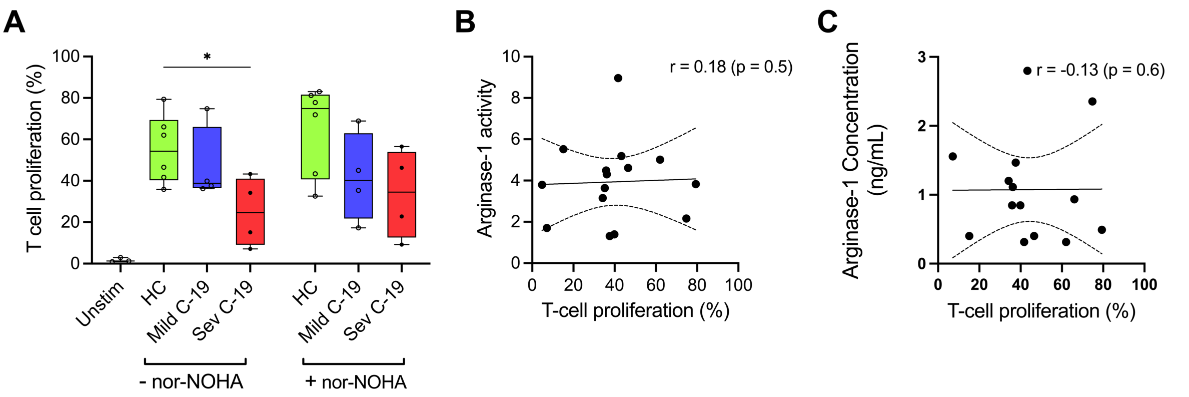

Figure S4: Neutrophil mediated T-cell suppression in severe patients is arginase-1 independent. Neutrophil derived supernatants were harvested from healthy controls (HC, n= 6), mild (n=4) and severe (n= 6) patients with COVID-19. (A) Rate of T cell proliferation upon co-culture with neutrophil supernatant in the absence (same as Figure 7A) or presence of the arginase 1 inhibitor nor-NOHA. Correlation of % T-cell proliferation with (B) arginase-1 activity (n=16) and (C) arginase-1 concentration (n=16) in neutrophil supernatants. Statistical analysis was performed using one-way ANOVA and spearman correlation. *p<0.05. Median with IQR is shown.

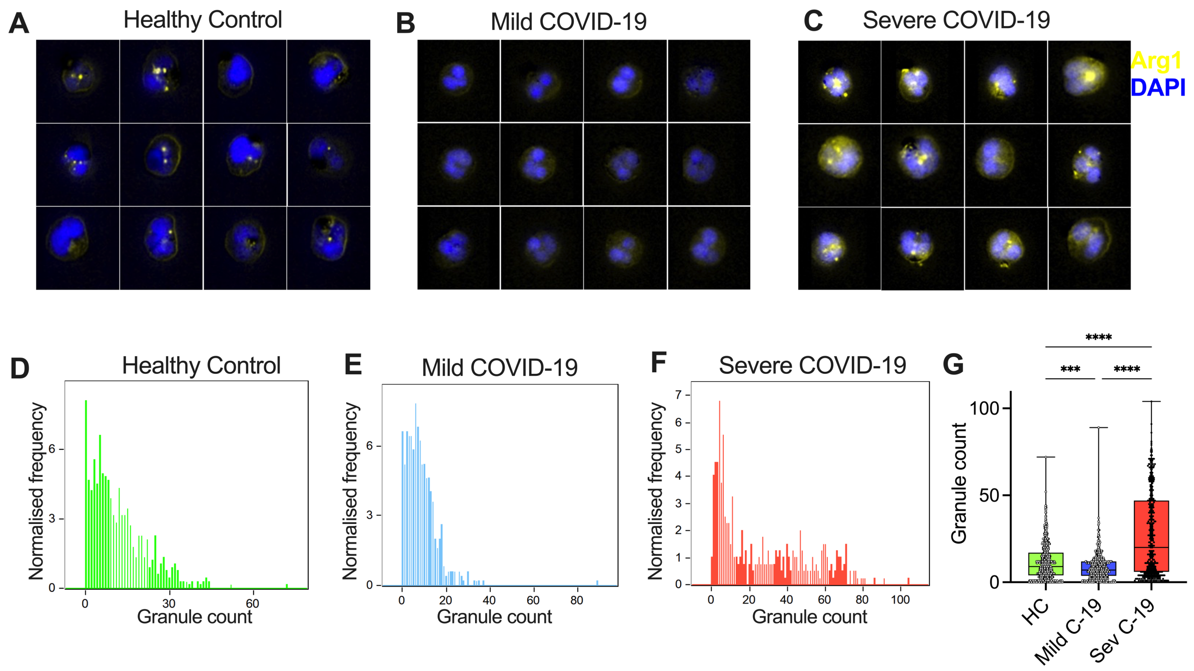

Figure S5. Imaging flow cytometry suggests aberrant intracellular arginase-1 expression in COVID-19 neutrophils. Fresh whole blood neutrophils were stained for intracellular arginase-1 and acquired on an imaging flow cytometer to determine the cellular localisation of arginase-1 in a healthy control and a patient with mild and severe COVID-19. Granule count was performed using the spot count feature of the IDEAS analysis software. Representative images of whole blood neutrophils from a (A) healthy control, (B) mild and (C) severe COVID-19 patient with DAPI stained nuclei (blue) and intracellular arginase-1 staining (yellow). Images were obtained at 60x magnification on ImageStream X MKII. Histograms showing arginase-1 granule count distribution in neutrophils of a (D) healthy control, (E) mild and (F) severe patient. (G) Plot of arginase-1 granule count from a healthy control, and a patient with mild and severe COVID-19. Each dot represents an individual cell. Statistical analysis was performed using Kruskal-Wallis test with Dunn's multiple comparisons test. ***$p<0.001$; ****$p<0.0001$. Median with IQR is shown.

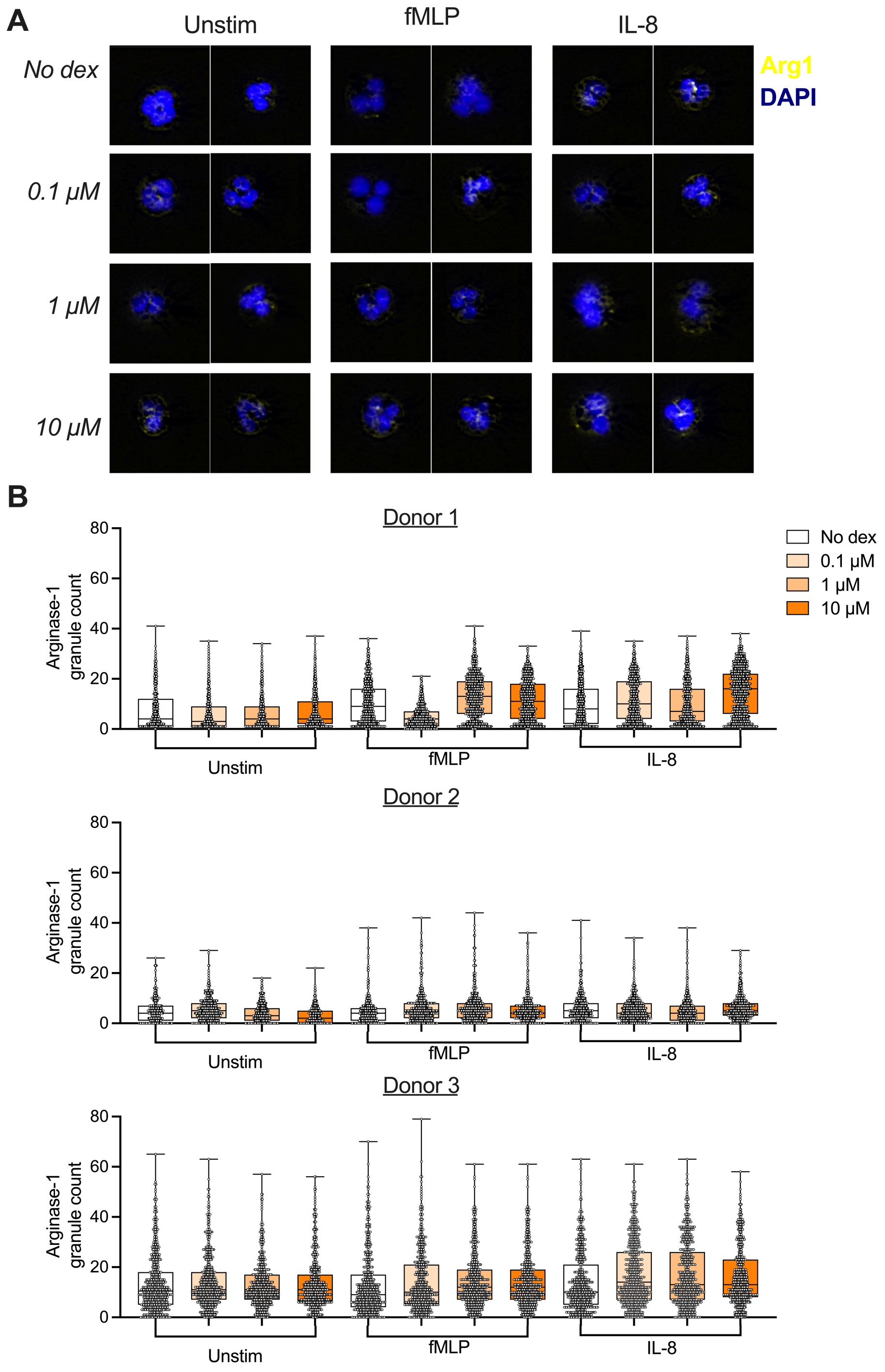

Figure S6. Dexamethasone treatment has no effect on intracellular arginase-1 expression pattern in healthy neutrophils. Neutrophils were isolated from healthy controls (n=3) and pre-treated with varying concentrations of dexamethasone 0, 0.1µM, 1µM, 10 µM for 4 hours. Cells were primed with 5 µg/mL cytochalasin B and left unstimulated or stimulated with 5 µg/mL fMLP or 200 ng/mL IL-8. **(A)** Representative images of neutrophils from each treatment condition with DAPI stained nuclei (blue) and intracellular arginase-1 staining (yellow) (n=3). **(B)** Arginase-1 granule count in unstimulated, fMLP or IL-8 stimulated cells from each donor(n=3). Each dot represents an individual cell.
